# Commentary: Navigating complexity. The role of cognitive load in guideline adherence among nurses

**DOI:** 10.1177/17449871251371975

**Published:** 2025-09-17

**Authors:** Sarah Bekaert

**Affiliations:** Senior Lecturer, Oxford Brookes University, UK

Commentary on: Mediator effects of cognitive load on relationship between task complexity and guideline adherence among clinical nurses: a cross-sectional survey of nurses in China (Zhang et al., 2025).

This robust study by Zhang et al. (2025) explores how mental effort, or cognitive load, influences the relationship between task complexity and nurses’ ability to follow clinical guidelines. Nurses often face complex tasks that demand both physical and mental effort. Certain challenges can make it harder to follow established care guidelines. For example, cognitive load, which refers to the mental effort needed to complete a task, can be influenced by how difficult the task is and how the information is presented. When tasks are too complex, the mental load may become overwhelming, making it harder for nurses to apply guidelines correctly. Understanding this relationship can help improve guideline use and, ultimately, the quality of patient care.

The study used three validated questionnaires to collect data. The first was a Guideline Adherence Questionnaire, which assessed how closely nurses followed clinical guidelines in their daily practice, focusing on nursing assessment, diagnosis, and intervention. The second was the Task Complexity Scale (Chinese version), a five-item tool that measured how complex nurses perceived their tasks to be, considering factors such as required knowledge, cooperation, and physical or mental effort. The third was the Measuring Different Types of Cognitive Load (MDT-CL), also in the Chinese version, a 10-item questionnaire which evaluated three forms of cognitive load: intrinsic, extraneous, and germane. Intrinsic cognitive load refers to the mental effort required to understand the inherent difficulty of a task; extraneous cognitive load to the unnecessary mental effort caused by poor design or distractions; and germane cognitive load to the mental effort invested in learning, processing, and applying new knowledge (Sweller et al., 2011). These tools helped the researchers assess the relationships between task complexity, cognitive load, and adherence to clinical guidelines. The authors are to be commended on the design of the study. Utilising objective measures to assess the psychological aspects of nursing work is essential for identifying stressors, and building clear evidence to call for effective systemic support strategies.

The study received 580 valid responses from clinical nurses. Power calculations suggested a sample of 200, which this study far exceeded. This suggested that this was a salient and important issue for nurses receiving the call to participate, hence the high response rate. Overall, nurses demonstrated a moderate level of adherence to clinical practice guidelines, with about one-quarter showing high adherence. Scores were consistent across different fields of nursing care, including assessment, diagnosis and intervention, suggesting universality across specific practice areas.

Analysis suggested that nurses who perceived their tasks as more complex were more likely to follow clinical guidelines. Drawing on Cognitive Load Theory (Sweller 1988; Sweller et al 2011), mental effort, also known as cognitive load, played a significant role in this relationship. Specifically, intrinsic cognitive load, which refers to the natural difficulty of the task and the mental effort required to understand it, was found to reduce guideline adherence. Similarly, extraneous cognitive load, which comes from distractions or poorly presented information (such as unclear instructions or inefficient processes), also had a negative impact. On the other hand, germane cognitive load, which reflects the mental effort invested in understanding, learning and applying useful knowledge, was linked to better guideline adherence. Showing that when nurses actively engaged with the information and tried to integrate it into their practice, they were more likely to follow the guidelines.

This study highlights that how nurses mentally process complex tasks, and specifically, the type of cognitive load they experience, directly affects their ability to follow clinical guidelines. Although complex tasks can overwhelm nurses by increasing intrinsic cognitive load, they can also enhance performance if germane cognitive load is stimulated, which supports understanding and application. The key message is that task complexity is not inherently negative; its impact depends on how it is managed cognitively. The authors recommend that to improve guideline adherence, nursing leaders should focus not only on reducing task difficulty but also on training, workflow design, and support strategies that lower unhelpful mental load and promote meaningful learning and engagement with guidelines.
